# Airway Malacia: Clinical Features and Surgical Related Issues, a Ten-Year Experience from a Tertiary Pediatric Hospital

**DOI:** 10.3390/children8070613

**Published:** 2021-07-20

**Authors:** Michele Ghezzi, Enza D’Auria, Andrea Farolfi, Valeria Calcaterra, Alessandra Zenga, Annalisa De Silvestri, Gloria Pelizzo, Gian Vincenzo Zuccotti

**Affiliations:** 1 Allergology and Pneumology Unit, V. Buzzi Children’s Hospital, 20154 Milan, Italy; enza.dauria@unimi.it (E.D.); andrea@farolfi.it (A.F.); alessandra.zenga@asst-fbf-sacco.it (A.Z.); 2 Department of Pediatrics, V. Buzzi Children’s Hospital, 20154 Milan, Italy; valeria.calcaterra@unipv.it (V.C.); gianvincenzo.zuccotti@unimi.it (G.V.Z.); 3 Pediatric and Adolescent Unit, Department of Internal Medicine, University of Pavia, 27100 Pavia, Italy; 4 Biometry & Clinical Epidemiology, Scientific Direction, Fondazione IRCCS Policlinico San Matteo, 27100 Pavia, Italy; a.desilvestri@smatteo.pv.it; 5 Department of Pediatric Surgery, V. Buzzi Children’s Hospital, 20154 Milan, Italy; gloria.pelizzo@asst-fbf-sacco.it; 6 Department of Biomedical and Clinical Science “L. Sacco”, University of Milan, 20157 Milan, Italy

**Keywords:** children, airway malacia, pediatric surgery

## Abstract

Background: Few studies have been carried out with the aim of describing the clinical course and follow-up of patients with tracheomalacia. We aim to describe the symptoms at diagnosis and the post-treatment clinical course of patients affected by airway malacia. Methods: We retrospectively analyzed characteristics of pediatric patients with a diagnosis of airway malacia. Patients were classified into three groups: bronchomalacia (BM), tracheomalacia (TM) and tracheo-bronchomalacia (TBM). Demographic and clinical data, diagnostic work-up and surgical treatment were recorded. Results: 13/42 patients were affected by congenital syndromes (30.9%). Esophageal atresia with or without tracheal-esophageal fistula (EA/TEF) was detected in 7/42 patients (16.7%). Cardiovascular anomalies were found in 9/42 (21.4%) and idiopathic forms in 13/42 (30.9%). BM occurred in 7/42 (16.6%), TM in 23/42 (54.7%) and TBM in 12/42 (28.6%). At the diagnosis stage, a chronic cough was reported in 50% of cases with a higher prevalence in EA/TEF (*p* = 0.005). Surgery was performed in 16/42 (40%) of children. A chronic cough and acute respiratory failure were correlated to the need for surgery. During follow-up, there was no difference in persistence of symptoms between conservative vs surgical treatment (*p* = 0.47). Conclusion: the management of tracheomalacia remains a challenge for pediatricians. Clinical manifestations, such as a barking cough and acute respiratory failure may suggest the need for surgery. Follow-up is crucial, especially in those patients affected by comorbidities, so as to be able to manage effectively the possible persistence of symptoms, including those that may continue after surgical treatment.

## 1. Introduction

During infancy, the tracheal cartilaginous framework is a crucial factor for maintaining airway patency [1]. Tracheomalacia (TM) is a condition of excessive tracheal collapsibility, due either to a disproportionate laxity of the pars membranacea or to compromised cartilage integrity, with consequent reduced tracheal lumen. When the main bronchi are also involved, the condition is defined as tracheobronchomalacia (TBM). Bronchomalacia (BM) represents a condition of excessive collapsibility of one or both of the main bronchi and is more commonly associated with congenital heart disease [2].

Primary or congenital airway malacia can be subdivided into either an isolated process or it can be associated with a condition or syndrome. The isolated primary tracheomalacia is usually benign because airway tone improves over time rather than progressively worsening. Secondary processes are related to extrinsic tracheal compression, such as from a vascular ring or mass, leading to progressive symptoms and morbidity. In addition, congenital abnormalities such as esophageal atresia with or without tracheal-esophageal fistula (EA/TEF) are often associated with tracheomalacia [3].

When a diagnosis is suspected, based on clinical history and physical examination, as reported by the recent ERS (European Respiratory Society) statement, a flexible bronchoscopy represents the “gold standard” diagnostic test. The degree of TM/TBM can be assessed either bronchoscopically or radiologically [4]. The patients affected by TM/TBM present with a wide spectrum of type and severity of symptoms and for this reason the evidence on detection, classification and management is poor. Moreover, there is no universally accepted classification of severity. Diagnosis of TBM can be delayed, especially in less severe forms. Observation and conservative management of symptoms are usually the preferred courses of action and they are based on chest physiotherapy and medical treatment, such as the use of bronchodilators and antibiotics, which are needed when cases are exacerbated. When medical treatment is not enough and patients remain unresponsive to it, surgery may be necessary to improve symptoms, both in non-severe and severe cases. [5]. Options include aortopexy or posterior tracheopexy to reduce external compression, tracheal resection of short affected tracheal segments, internal stents to limit tracheal collapse and external airway splinting.

Few studies have been done with the aim of investigating the clinical features and follow-up of patients affected by this condition. Those few studies have a paucity of data on symptoms post-surgical treatment.

This study aims to describe the symptoms at diagnosis and the clinical course in our cohort of patients affected by TM/TBM or BM, including both those conservatively and surgically managed.

## 2. Materials and Methods

### 2.1. Patients

We retrospectively reviewed the records of pediatric patients (≤18 years) who were diagnosed with airway malacia and were subsequently followed at the Pediatric Pulmonology Unit of the Children’s Hospital V. Buzzi, Milano, Italy, from January 2010 to December 2019.

According to the level of the airway malacia, patients were classified into three groups:

- Subjects with bronchomalacia (BM).
- Subjects with tracheomalacia (TM).
- Subjects with tracheo-bronchomalacia (TBM).

All data were collected from the medical records of patients diagnosed with airway malacia and these data included demographic characteristics, clinical history, imaging studies, bronchoscopy findings, surgical treatment, medications and outcomes.

The current retrospective study protocol was approved by the Local Institutional Review Board. Informed consent was not obtained as this was a retrospective study; however, anonymity of the patients was preserved.

### 2.2. Data Collection

#### 2.2.1. Clinical Data

Clinical data included: age, gender, presence of associated anomalies or pathologies and symptoms at the diagnosis and during the follow-up stages. In particular, we recorded the presence at the onset of the disease of a chronic cough, recurrent respiratory infections, recurrent pneumonia, dysphagia, reduced exercise tolerance and/or an exercise-induced cough, cyanosis, acute respiratory failure, stridor and/or neonatal respiratory distress. 

According to the ERS statement, there is no universally accepted classification. Considering the conditions associated with TM and TBM, the affected patients were classified as follows: congenital syndromes, significant and complex comorbidities, EA/TEF not associated with other congenital disorders and cardiovascular anomalies and idiopathic forms.

#### 2.2.2. Diagnostic Work-up and Surgery

The use of a CT thorax and/or a flexible bronchoscopy as a diagnostic evaluation was recorded. 

A multidetector CT was performed with an intravenous contrast agent, and, ideally, the examination was performed with breath holding at suspended inspiration. A bronchoscopy was performed under sedation, introducing the fiberoptic bronchoscope through a face mask. During spontaneous breathing, the larynx, trachea and bronchial systems were visualized in order to detect any anomalies. The presence and degree of tracheal narrowing, in inspiration and expiration, were evaluated from video recordings.

According to the degree of the TM/TBM, malacia was described as mild (50–75% reduction), moderate (75–90%) or severe (>90%), as established by the recent ERS statement [4]. The types of surgical approach and post-operative outcome were also recorded.

### 2.3. Statistical Analysis

All analyses were performed using Stata 16 (StataCorp, College Station, TX, USA). Data were described with the mean, standard deviation (SD), if continuous and as counts and percent if categorical. Comparisons between groups were made with a one-way analysis of variance. The association of categorical variables was assessed with chi square or the Fisher’s exact test. The association between the need for surgery and clinical features or symptoms was evaluated fitting logistic regression models. All tests were two-sided. A *p*-value < 0.05 was considered statistically significant.

## 3. Results

### 3.1. Demographic and Clinical Features of Patients

Of the 42 patients included in the study, 21 were female and 21 male (*p* = 0.9). The mean age at diagnosis was 2.1 ± 2.6 years (range 0–10.7 years). 

According to the level of airway malacia, TM occurred in 23/42 patients (54.7%), BM in 7/42 (16.6%) and TBM in 12/42 (28.6%), with no significant difference between sexes (*p* = 0.6).

In total, 13/42 patients were affected by congenital syndromes associated with malacia (30.9%), EA/TEF that was not associated with other congenital disorders was detected in 7/42 patients (16.7%), cardiovascular anomalies in 9/42 (21.4%) and idiopathic forms in 13/42 (30.9%), with no difference in males compared to females (*p* = 0.8).

Patient characteristics and onset symptoms are reported in Table 1.

### 3.2. Symptoms

As reported in Table 1, at diagnosis, a chronic cough was reported in 50% of cases with no difference between sexes (*p* = 0.2) and airways malacia level (*p* = 0.26). A cough was more frequent in children with EA/TEF compared to other pathogenetic groups (*p* = 0.005).

The overall prevalence of recurrent respiratory infections was 57.4%, with equal distribution in BM, TM and TBM (*p* = 0.6). A clinical presentation with recurrent respiratory infections was more prevalent in patients with congenital syndromes (71.4%) and in patients with EA/TEF (69.23%) compared to other groups (44.4% in cardiovascular anomalies and 16.7% in idiopathic forms, respectively), without, however, reaching any statistical significance (*p* > 0.05), as well as recurrent pneumonia (*p* = 0.123).

The reduced exercise tolerance and/or an exercise-induced cough were significantly more frequent in patients with BM (42.8%) compared to TM (0%) and TBM (16.6%), *p* = 0.008, Table 1.

The prevalence of dysphagia was similar between BM, TM and TBM as well as according to the pathogenesis. Interestingly, stridor, cyanosis and neonatal respiratory distress were reported only in patients with TM or TBM, even if this was of no statistical significance. Furthermore, stridor was more frequent in patients with cardiovascular anomalies and idiopathic forms (46.7%).

### 3.3. Diagnostic Work-Up

A thorax CT scan was performed in 34/42 (80.9%) of patients and the result was diagnostic in 91% of cases, with no significant difference between BM, TM and TBM (*p* = 0.3). 

In 36/42 (85.7%) of patients, a flexible bronchoscopy was performed and it was diagnostic in all cases. 

Among our patients, 33.3% of them presented with a severe form of malacia.

### 3.4. Surgical Treatment

Surgical treatment was performed in 16/42 (40%) of children, with no difference in males compared to females (*p* = 0.8). The need for surgery was no different between BM, TM and TBM (*p* = 0.9) and among different conditions associated with malacia (*p* = 0.22).

As reported in Table 2, aortopexy was the most frequent surgical intervention (7/16, 43.7%); 1 patient (6.2%) required tracheoplasty and 3 patients (18.7%) tracheostomy; 3 patients (18.7%) underwent surgical repair of a congenital double aortic arch, while 2 patients (12.5%) affected by BM required internal stenting. 

Clinical presentation with a chronic cough and acute respiratory failure was significantly related to the need for surgery (*p* = 0.03 and *p* = 0.04, respectively). No other significant associations between onset symptoms and need for surgery were detected. Particularly, the degree of severity of TM/TBM was not related to surgical treatment (*p* = 0.3).

### 3.5. Symptom Persistence after Conservative or Surgical Treatment 

Follow-up duration was almost 2 years. Persistence of a typical barking cough, though less intense than that at onset, was the most frequent symptom cited at follow-up, with a prevalence of 45.2%. Other reported symptoms were: recurrent respiratory infections (>3/year: 21.4%) and reduced exercise tolerance (16.7%). Only one patient presented with chronic respiratory failure. 

The overall prevalence of persistent symptoms during follow-up was 54.8%, with no difference in patients submitted to surgery compared to the others (*p* = 0.47). 

In Table 3, the persistence of symptoms according to the need for surgery, airway malacia levels and lesion severity are reported. The level of the airway malacia and the severity of the lesion are not correlated to post-surgical complications (*p* = 0.6 and *p* = 0.08, respectively).

## 4. Discussion

We reviewed a 10-year single-center experience of pediatric airway malacia and its management. 

BM, TM and TBM may be primary abnormalities of the large airways or they could be due to a secondary or acquired cause. As reported, we did not find any differences in the prevalence of these conditions between males and females [6]. In agreement with the data, we found that isolated BM is a relatively rare condition [7,8] and that TM/TBM may be frequently associated with genetic syndromes and esophageal atresia repairs [3,4,9]. 

The mean age at diagnosis of more than 2 years in the patients we studied supports the data that, while severe malacia may be evident from birth, most patients typically exhibit symptoms later, resulting in a delay in diagnosis and in treatment [7,10,11]. 

In most of our patients, the diagnosis, as well as the measurement of the tracheal lumen reduction, were established by fibrobronchoscopy, the gold standard exam according to the ERS statement [4]. A total of 33% of our patients were affected by a severe form. Most of them also underwent a chest CT scan in order to confirm secondary tracheomalacia, due to vascular malformations and/or to evaluate the lung parenchyma. 

According to the data, in our study, a chronic cough and recurrent respiratory infections were the most common symptoms detected at the diagnosis, independent of the level of the airway malacia. In a recent Italian cohort of children affected by a chronic cough, in 32% of cases, tracheomalacia, due to an aberrant innominate artery, was found [12]. Boogaard et al. described symptoms in 96 outpatients with primary airway malacia and without comorbidities: a cough was reported in 83% of patients [13]. On the contrary, the prevalence of a chronic barking cough was lower in our patients than that reported by Boogard et al. [13], probably due to the most frequent presentation of recurrent respiratory infections and recurrent pneumonia among patients with TM associated with genetic syndromes or esophageal atresia repairs [3]. 

Among our patients, other symptoms, such as reduced exercise tolerance and/or exercise-induced cough, seemed to be more prevalent in patients with BM compared to TM and TBM. 

We reported that the clinical presentation of a chronic barking cough and acute respiratory failure was significantly related to the need for surgery. Similar to our results, Okata et al. found that patients with episodes of acute life-threating events have a higher risk of surgical intervention [14].

Despite the fact that the presence of a barking cough in the diagnosis of tracheomalacia has shown high specificity [15], our results may underline the importance of close monitoring when these symptoms occur. Further studies are essential in order to establish a correlation between this symptom and the need for surgery. 

Even though recent recurrent infections have been suspected as factors related to the need for surgery [10], we did not observe any correlation between recurrent infections and the need of surgery.

In line with the study by Masters [16], our study also established that neither the sites nor the severity of malacia were correlated to the severity of illness and/or the persistence of symptoms during follow-up. 

Forty percent of our patients underwent a surgical intervention, and aortopexy was the most frequent type of procedure [5,17]. We did not observe a difference in prevalence of symptoms during follow-up between patients who underwent surgery and patients managed with a conservative treatment, as reported by Rossi et al. [18]. It is likely that this lack of difference has in turn led to the lack of general consensus regarding the criteria for choosing surgery.

In patients without comorbidities, an improvement of symptoms 1–2 years after post-surgical treatment has been reported [19,20]. In contrast, among our patients who underwent surgical treatment, symptoms persisted after treatment in 47% of patients. These data could be explained by a higher proportion of patients affected by genetic syndromes with other comorbidities or EA/TE than in other studies. In fact, in one of the few series published, with a proportion of patients affected by comorbidities, similar to that of our study, a higher susceptibility to respiratory infections was also reported [21]; Vazquez-Jimenez et al. described the same findings in children who underwent aortopexy [22]. 

The expression of cytokines, IL-8, IL17A, and IL-1α, is higher in patients affected by tracheomalacia, and this has the potential to indicate the presence of chronic airway inflammation and also lung microbiota disequilibrium, with a prevalence of Pseudomonas at the BALF [23]. These mechanisms are probably linked to the persistence of symptoms, especially respiratory exacerbations during follow-up in patients with comorbidities. 

In contrast to what was reported in a small cohort by Zeng Hao Wong et al. [24] and in the study by Rijnberg et al. evaluating 100 patients who underwent aortopexy [25], in our patients, we did not find a correlation between bronchus involvement and symptom persistence after surgical treatment, but other surgical details could explain this difference. 

We recognize that this study has some limitations: first of all, the small number of patients, which makes it difficult to highlight any significant differences in clinical presentation, management and outcome between the different categories of patients; furthermore, the retrospective aspect of the study does not allow any definitive conclusions to be drawn on the best approach, surgical or conservative, for the patients. Moreover, a longer follow-up period could give further information on long-term outcomes in these patients. Additionally, further larger studies are key in order to confirm a correlation between a typical clinical symptom, such as a barking cough, not only with the diagnosis of TM, as already reported, but also with the need for surgery. On the other hand, our study highlights the importance of the follow-up, especially in those patients affected by genetic syndromes or with comorbidities, who may have persistent symptoms even after surgery, more than those who are not affected.

## 5. Conclusions

The management of tracheomalacia remains a challenge for pediatricians. Clinical manifestations, such as a barking cough and acute respiratory failure, may suggest the need for surgery. Early diagnosis is important so as to allow for prompt surgical or medical intervention; follow-up is also crucial, especially in those patients affected by comorbidities, in order to be able to manage possible persistence of symptoms, after surgical treatment. As symptoms are varied and non-specific, early recognition and close monitoring of both surgically and conservatively managed patients are mandatory. 

## Figures and Tables

**Table 1:** Patient characteristics and onset symptoms according to the level of the airway malacia.

	Bronchomalacia *n* = 7	Tracheomalacia *n* = 23	Tracheo-Bronchomalacia *n* = 12	*p*
Age	2.52 (2.40)	1.53(1.91)	2.97(3.60)	0.29
Sex (F/M)	3/4	13/10	5/7	0.6
Cough *n* (%)	5 (71.4%)	12 (52.2%)	4 (33.3%)	0.26
Recurrent Respiratory Infections *n* (%)	5 (71.4%)	12 (52.2%)	7 (58.3%)	0.66
Dysphagia *n* (%)	0 (0%)	1 (4.3%)	0 (0%)	0.65
Pneumonia *n* (%)	1 (14.3%)	4 (17.4%)	2 (16.7%)	0.98
Reduced Exercise Tolerance and/or an Exercise-Induced Cough *n* (%)	3 (42.8%)	0 (0%)	2 (16.7%)	0.008
Cyanosis *n* (%)	0 (0%)	3 (13.0%)	1 (8.3%)	0.58
Stridor *n* (%)	0 (0%)	6 (26.1%)	5 (41.7%)	0.13
Acute Respiratory Failure *n* (%)	1 (14.3%)	5 (21.7%)	2 (16.7%)	0.88
Neonatal Respiratory Distress *n* (%)	0 (0%)	2 (8.7%)	1 (8.3%)	0.72

**Table 2:** Surgical treatment according to the level of the airway malacia and associated conditions.

Type of Surgery	Level of the Airway Malacia			Conditions Associated with Airway Malacia		
	BM	TBM	TM	EA/TEF	Cardiovascular Anomalies	Syndromic Patients
Aortopexy	0	3 (42.9%)	4 (57.1%)	2	1	4
(*n* = 7)				(28.6%)	(14.3%)	(57.1%)
Double Aortic Arch	0	0	3 (100%)	0	3	0
(*n* = 3)					(100%)	
Tracheoplasty	0	0	1 (100%)	0	0	1
(*n* = 1)						(100%)
Tracheal Stenting	2 (100%)	0	0	0	2	0
(*n* = 2)					(100%)	
Tracheostomy	0	2 (66.7%)	1 (33.3%)	0	2	1
(*n* = 3)					(66.7%)	(33.3%)

Abbreviation: BM: bronchomalacia; TBM: tracheo-bronchomalacia; TM: tracheomalacia; EA/TEF: esophageal atresia with or without tracheal-esophageal fistula.

**Table 3:** The prevalence of symptoms according to the need for surgery, airway malacia level and airway malacia grade.

Variables	LTRI > 3/years	Cough	Reduced Exercise Tolerance	Chronic Respiratory Failure
	*p*	*p*	*p*	*p*
	%	%	%	%
Surgical Treatment	*p* = 0.62	*p* = 0.28	*p* = 0.12	*p* = 0.22
—yes	24.0%	52.0%	24.0%	0%
—no	17.65%	35.29%	5.88%	5.88%
Level of the Malacia	*p* = 0.20	*p* = 0.78	*p* = 0.62	*p* = 0.27
- Bronchomalacia	30.43%	43.48%	13.04%	0%
- Tracheomalacia	0%	57.14%	28.57%	0%
- Tracheobronchomalacia	16.67%	41.67%	16.67%	8.3%
Malacia Degree	*p* = 0.12	*p* = 0.09	*p* = 0.42	*p* = 0.35
- Mild	33.33%	61.9%	23.81%	0%
- Moderate	0%	28.57%	14.29%	0%
- Severe	14.29%	28.57%	7.14%	7.14%

Abbreviation: LTRI: low tract respiratory infections.

## Data Availability

The data presented in this study are available on request from the corresponding author.
